# Analysis of the EGFR Amplification and CDKN2A Deletion Regulated Transcriptomic Signatures Reveals the Prognostic Significance of *SPATS2L* in Patients With Glioma

**DOI:** 10.3389/fonc.2021.551160

**Published:** 2021-04-20

**Authors:** Haiwei Wang, Xinrui Wang, Liangpu Xu, Ji Zhang, Hua Cao

**Affiliations:** ^1^Medical Research Center, Fujian Maternity and Child Health Hospital, Affiliated Hospital of Fujian Medical University, Fuzhou, China; ^2^State Key Laboratory for Medical Genomics, Shanghai Institute of Hematology, Rui-Jin Hospital Affiliated to School of Medicine, Shanghai Jiao Tong University, Shanghai, China

**Keywords:** glioblastoma, lower grade glioma, EGFR amplification, CDKN2A deletion, *SPATS2L*, the cancer genome atlas

## Abstract

**Purpose:** This study was conducted in order to analyze the prognostic effects of epidermal growth factor receptor (EGFR) and CDKN2A alterations and determine the prognostic significance of EGFR and CDKN2A alterations on regulated genes in patients with glioblastoma (GBM) or lower grade glioma (LGG).

**Methods:** The alteration frequencies of EGFR and CDKN2A across 32 tumor types were derived from cBioPortal based on The Cancer Genome Atlas (TCGA) datasets. The Kaplan–Meier analysis was used to determine the prognostic significance of EGFR and CDKN2A alterations. EGFR and CDKN2A alterations on regulated expression signatures were identified from RNA-seq data in the TCGA GBM datasets. The prognostic significance of EGFR and CDKN2A alterations on regulated genes in patients with glioma was determined using the TCGA and the Chinese Glioma Genome Atlas (CGGA) datasets.

**Results:** Compared with the other 31 tumor types, EGFR amplification and CDKN2A deletion particularly occurred in patients with GBM. GBM patients with EGFR amplification or CDKN2A deletion demonstrated poor prognosis. Statistical analysis showed the coexistence of EGFR alteration and CDKN2A deletion in GBM patients. We identified 864 genes which were commonly regulated by EGFR amplification and CDKN2A deletion, and those genes were highly expressed in brain tissues and associated with the cell cycle, EBRR2, and MAPK signaling pathways. *Spermatogenesis-associated serine-rich 2-like gene* (*SPATS2L*) was upregulated in GBM patients with EGFR amplification or CDKN2A alteration. Higher expression levels of *SPATS2L* were associated with worse prognosis in patients with GBM in both TCGA and CGGA datasets. Moreover, the expression levels of *SPATS2L* were higher in patients with a mesenchymal subtype of GBM. Statistical analysis also showed that the coexistence of EGFR alteration and CDKN2A deletion was significant in patients with LGG. *SPATS2L* was upregulated in LGG patients with EGFR amplification or CDKN2A alteration. Furthermore, higher expression levels of *SPATS2L* were associated with worse prognosis in patients with LGG in both TCGA and CGGA datasets. The expression levels of *SPATS2L* were higher in patients with an astrocytoma subtype of LGG. Finally, the coexistence and unfavorable prognostic effects of EGFR amplification and CDKN2A alteration were validated using the Memorial Sloan Kettering Cancer Center (MSKCC) glioma datasets.

**Conclusions:** EGFR amplification and CDKN2A deletion of the regulated gene *SPATS2L* have significant prognostic effects in patients with GBM or LGG.

## Introduction

Glioma is the most common type of brain malignancy in adults and is one of the leading causes of cancer-related death (1). Glioma is a heterogeneous disease and is divided into different subtypes based on histological characteristics and grades (2). Lower grade glioma (LGG) is grade II–III glioma and glioblastoma (GBM) is grade IV glioma (3). GBM and LGG demonstrate different clinical outcomes and molecular profiling (4, 5). Compared with LGG, GBM patients have worse prognosis (6). Even with standard temozolomide or radiation treatment, the median survival of patients with GBM is only 12.6 months, representing one of the most aggressive types of cancer (7). Gene expression (8, 9), DNA methylation profiling (10), microRNA signature (11, 12), and immune-related lncRNA signature (13) are used as prognostic biomarkers in patients with glioma; however, new effective prognostic biomarkers are needed. In the present study, we tried to analyze the prognostic significance of epidermal growth factor receptor (EGFR) amplification and CDKN2A alteration on regulated genes in patients with glioma.

EGFR is a trans-membrane receptor tyrosine kinase, belonging to the ERBB family (14). The molecular landscape has detected EGFR gene alterations in more than half of patients with GBM (15). EGFR signaling is required to sustain the uncontrolled proliferation in tumor initiation and progression of GBM (16, 17). EGFR gene alteration is associated with the therapy response and clinical survival of GBM patients (18). Activating the EGFR signaling pathway in lung or colon cancer patients confers the sensitivity of EGFR inhibitors (19–21). However, GBM tumor cells usually maintain EGFR signaling activity even under EGFR inhibition treatment (22), and strategies targeting EGFR have failed in clinical trials (23). Those results suggest the complex regulatory networks of EGFR in GBM and the functions of EGFR still need extensive studies (24).

CDKN2A is a driver tumor suppressor gene, which regulates cell cycle progress by cyclin-dependent kinases CDK4 and CDK6 (25). The loss of CDKN2A promotes glioma formation and tumor metastasis (26, 27). CDKN2A deletion is associated with the classification and clinical outcomes of glioma (28). Interestingly, CDKN2A and EGFR are converged on cell cycle regulation and both are altered in the early stage of glioma development (29). CDKN2A deletion is part of the mechanism mediated by EGFR inhibitor resistance (30). In some lung cancer patients, CDKN2A deletion and EGFR mutation are coexistent to mediate poor drug response (31, 32). However, the connections between CDKN2A and EGFR in glioma are unclear.

*Spermatogenesis-associated serine-rich 2-like gene* (*SPATS2L*) is ubiquitously expressed in multiple tissues (33). Genome-wide association analysis reveals the functions of *SPATS2L* in asthma development (34). However, the functions of *SPATS2L* in cancer development are almost never reported. Also, the prognostic effects of *SPATS2L* in glioma and the associations between *SPATS2L* and EGFR and CDKN2A alterations are unknown. In the present study, using The Cancer Genome Atlas (TCGA), the Chinese Glioma Genome Atlas (CGGA), the Memorial Sloan Kettering Cancer Center (MSKCC), and Gene Expression Omnibus (GEO) datasets, the prognostic significance of EGFR and CDKN2A alteration was determined. We also found that EGFR amplification and CDKN2A deletion of the regulated gene *SPATS2L* had significant prognostic effects in patients with GBM or LGG.

## Materials and Methods

### Data Collection

The TCGA gene expressions along with the clinical datasets were downloaded from the TCGA hub (https://tcga.xenahubs.net). The CGGA datasets are available at the http://www.cgga.org.cn/index.jsp website. The gene expression series matrix of glioma tissues was downloaded from the GEO website (www.ncbi.nlm.nih.gov/geo), including the GSE4412, GSE83300, GSE16011, GSE43378, GSE83294, and GSE16011 datasets.

### OncoPrint of EGFR and CDKN2A Alterations

The genomic alterations of EGFR and CDKN2A across 32 types of tumor patients or in glioma patients were downloaded from cBioPortal (version 3.2.0) based on the TCGA and MSKCC datasets (http://www.cbioportal.org/index.do).

### TCGA Data Processing

GBM gene expression profile was analyzed using the TCGA GBM RNA-seq datasets. The differentially expressed genes between GBM patients with EGFR or CDKN2A alteration and GBM patients without EGFR or CDKN2A alteration were determined using paired Student's *t*-test. Genes with *P* < 0.01 were chosen to be significantly different.

### GEO Data Processing

The GEO expression datasets were annotated with corresponding probe sets and processed using the “plyr” package (version 1.8.5) in R software (version 3.5.0, https://www.r-project.org/). The plyr package was used for splitting, applying, and combining data and could be downloaded from Bioconductor (https://cran.r-project.org/web/packages/plyr/index.html).

### Heatmap Presentation

Heatmaps were created using the R software “pheatmap” package (version 1.0.12). The “pheatmap” package and the basic usage were downloaded from Bioconductor (https://cran.r-project.org/web/packages/pheatmap/). The clustering scale was determined by the “average” method. The clustering distance was determined by the “correlation” method.

### Survival Analysis

Kaplan–Meier plots were created using the “survival” package (version 3.1-8) in R statistics software (https://cran.r-project.org/web/packages/survival/index.html). GBM or LGG patients were divided into two clusters based on the mean expression levels of *SPATS2L* or alterations of EGFR or CDKN2A. The Kaplan–Meier estimator was applied to determine the clinical outcomes of the different clusters of glioma patients. *P*-values were determined using the log-rank test.

### Kyoto Encyclopedia of Genes and Genomes Signaling Pathway Enrichment Analysis

KEGG signaling pathways and tissue-specific expression were performed using the Database for Annotation, Visualization and Integrated Discovery (DAVID) tool (version 6.8; https://david.ncifcrf.gov). DAVID is a functional annotation tool for a list of genes. Enrichment results with *P* < 0.05 were considered to be statistically significant.

### Statistical Analysis

The box plots were generated from GraphPad Prism software (version 5.0; GraphPad Software, Inc.). Statistical analysis was performed using two-tailed paired Student's *t*-test in R software. A *P* < 0.05 was chosen to be significantly different.

## Results

### Alteration Frequencies and Prognostic Relevance of EGFR Amplification or CDKN2A Deletion Across 32 Tumor Types

**Figure 1:**
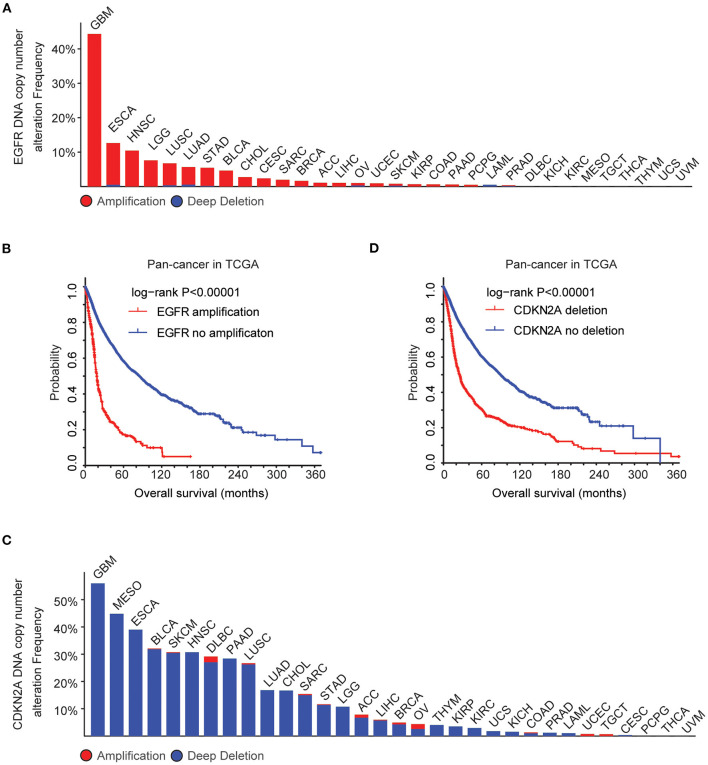
Alteration frequencies and prognostic relevance of epidermal growth factor receptor (EGFR) alteration and CDKN2A deletion across 32 tumor types. **(A)** Percentage of cancer patients with EGFR DNA copy number alterations across 32 tumor types. Red indicated EGFR amplification and blue indicated EGFR deletion. **(B)** The Kaplan–Meier plot demonstrated the prognostic effect of EGFR DNA copy number alterations across 32 tumor types of patients in The Cancer Genome Atlas (TCGA) datasets. The log-rank test was used to determine the different overall survival between patients with (red) or without (blue) EGFR amplification. **(C)** Percentage of cancer patients with CKDN2A DNA copy number alterations across 32 tumor types. **(D)** The Kaplan–Meier plot demonstrated the different overall survival of patients with (red) or without (blue) CKDN2A deletion across 32 tumor types. *P*-values were generated from the log-rank test.

Using cBioPortal (35, 36), the biological relevance of EGFR alteration in tumor patients derived from the TCGA Pan-cancer datasets was determined. Across 32 different tumor types, the frequency of EGFR amplification was the highest in patients with GBM (Figure 1A). More than 40% GBM patients had EGFR amplification. Moreover, tumor patients with EGFR amplification demonstrated worse prognosis compared with patients without EGFR amplification (*P* < 0.0001) (Figure 1B), suggesting that EGFR amplification was a biomarker correlated with the clinical outcomes across different tumor types. EGFR signaling was also activated by a specific EGFR mutation. EGFR mutation also particularly occurred in patients with GBM. More than 25% GBM patients had EGFR mutations (Supplementary Figure 1A). Similarly, tumor patients with EGFR mutations demonstrated worse prognosis compared with patients without EGFR mutations (*P* < 0.0001) (Supplementary Figure 1B).

GBM patients also had the highest frequency of CDKN2A deletion. More than 50% GBM patients had CDKN2A deletion (Figure 1C), and CDKN2A deletion was a bad prognosis across different tumor types (Figure 1D). Those results showed that EGFR amplification, EGFR mutation, and CDKN2A deletion particularly happened in patients with GBM and those genomic alterations may determine the bad prognosis of GBM.

### Coordinated Prognostic Relevance and Co-occurrence of EGFR Amplification and CDKN2A Deletion in Patients With GBM

**Figure 2:**
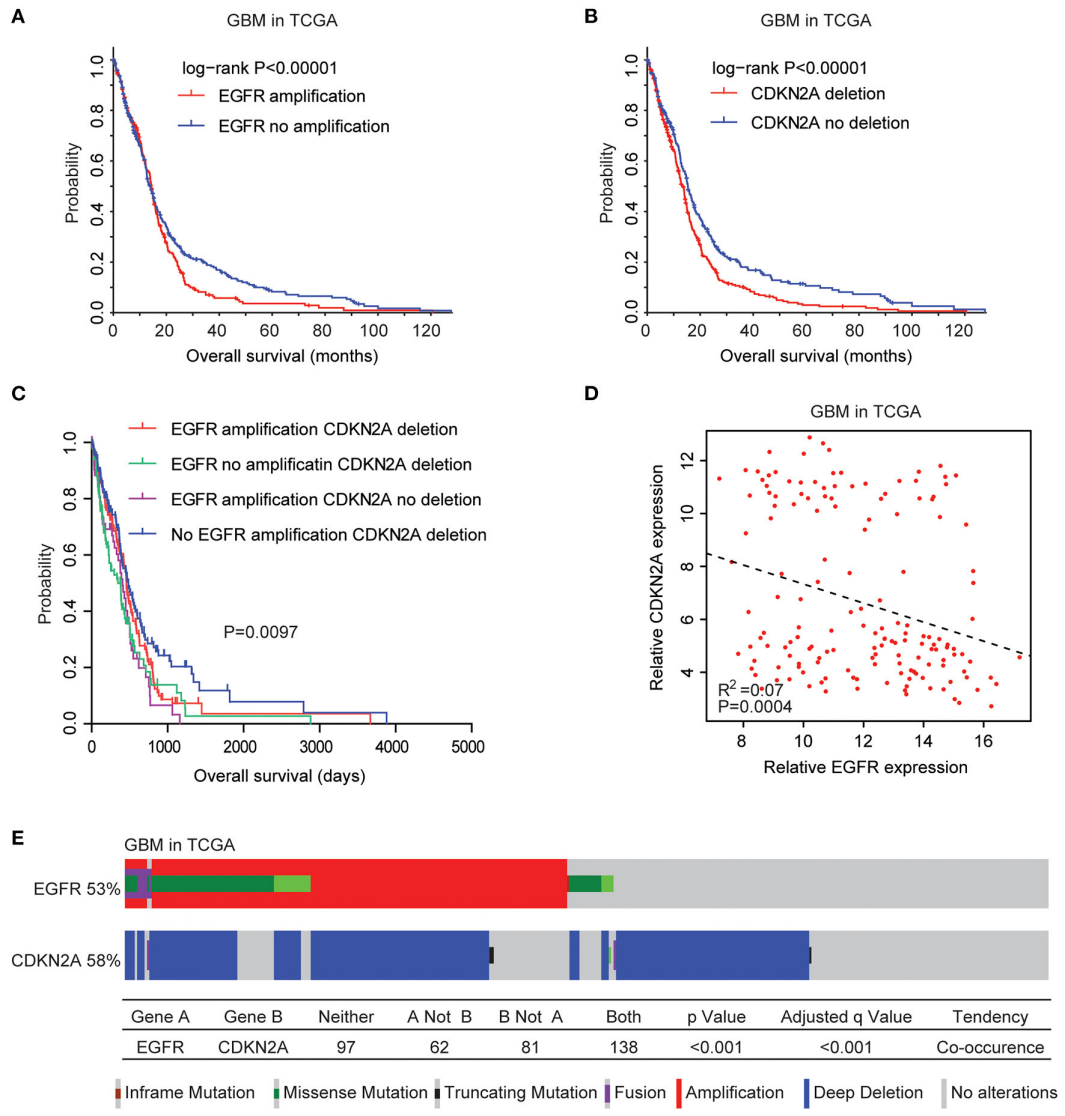
Coordinated prognostic relevance and co-occurrence of EGFR alteration and CDKN2A deletion in patients with glioblastoma (GBM). **(A)** The Kaplan–Meier plot demonstrated the prognostic effect of EGFR amplification in patients with GBM in the TCGA datasets. The log-rank test was used to determine the different overall survival of GBM patients with (red) or without (blue) EGFR amplification. **(B)** The Kaplan–Meier plot demonstrated the different overall survival of GBM patients with (red) or without (blue) CKDN2A deletion. *P*-values were generated from the log-rank test. **(C)** The Kaplan–Meier plot demonstrated the different overall survival of GBM patients with different alterations of EGFR and CDKN2A. **(D)** Spearman correlations of EGFR and CDKN2A expression in GBM patients. **(E)** OncoPrint demonstrated the co-occurrence of EGFR alteration and CDKN2A deletion in patients with GBM derived from the TCGA dataset. Red indicated gene amplification, blue indicated gene deletion, and green represented gene mutation. Each line represented one patient.

We then tested the prognostic effects of EGFR amplification, EGFR mutation, and CDKN2A deletion in patients with GBM. GBM patients with EGFR amplification had worst prognosis than patients without EGFR amplification (*P* < 0.0001) (Figure 2A). GBM patients with CDKN2A deletion also demonstrated worse prognosis compared with patients without CDKN2A deletion (*P* < 0.0001) (Figure 2B). However, there was no difference in clinical overall survival between GBM patients with or without EGFR mutation (*P* = 0.98) (Supplementary Figure 1C). Furthermore, the expression levels of EGFR and CDKN2A were not associated with the clinical outcomes of patients with GBM (Supplementary Figure 2).

Moreover, GBM patients without EGFR amplification and CDKN2A deletion had the best prognosis than GBM patients with EGFR amplification or CDKN2A deletion or with both alterations (Figure 2C). Also, Spearman correlation demonstrated negative correlations of EGFR and CDKN2A expression in the TCGA GBM datasets (Figure 2D). All those results emphasized the synergetic effects of EGFR amplification and CDKN2A deletion in determining the clinical outcomes of GBM patients.

Genetically, EGFR alteration and CDKN2A deletion were also connected. Among the 378 GBM patients in the TCGA datasets, 53% of the patients had EGFR alteration and 58% of the patients had CDKN2A deletion. Interestingly, 138 (36%) GBM patients had both EGFR and CDKN2A alterations. Statistical analysis showed that the coexistence of EGFR alteration and CDKN2A deletion was significant (*P* < 0.0001) (Figure 2E).

### The Expression Signature Regulated by EGFR Amplification and CDKN2A Deletion in Patients With GBM

**Figure 3:**
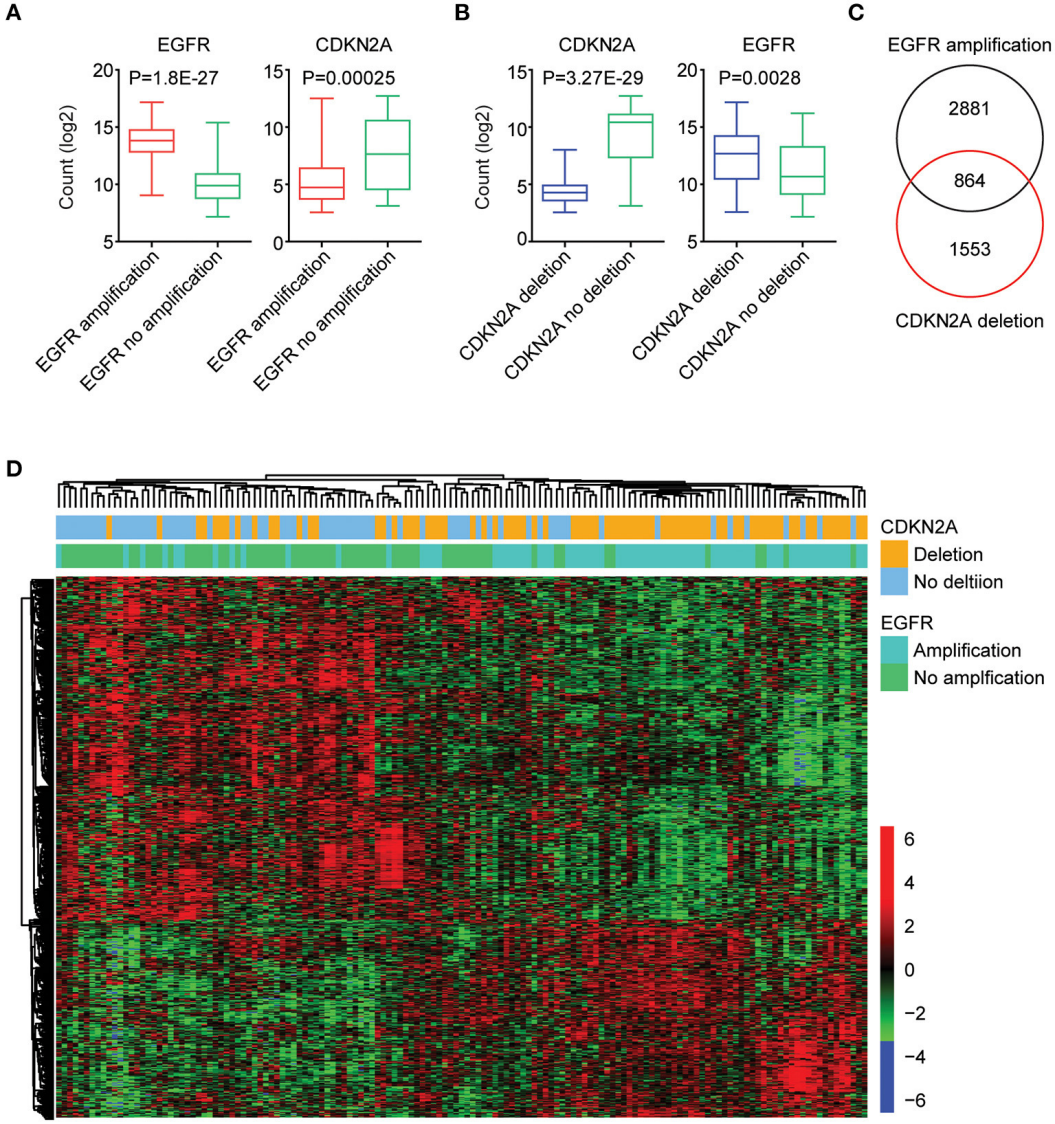
The expression signature regulated by EGFR amplification and CDKN2A deletion in patients with GBM. **(A)** Box plots showed the EGFR and CDKN2A expression levels (log_2_ normalization count) in TCGA GBM patients with (red) or without (green) EGFR amplification. **(B)** Box plots showed the EGFR and CDKN2A expression levels (log_2_ normalization count) in TCGA GBM patients with (blue) or without (green) CDKN2A deletion. **(C)** EGFR amplification and CDKN2A deletion on regulated genes (*P* < 0.01) in GBM patients were identified. The Venn diagram depicted the number of commonly regulated genes by EGFR amplification and CDKN2A deletion. **(D)** Unsupervised clustering heatmap demonstrated the commonly regulated genes by EGFR amplification and CDKN2A deletion.

Next, we further tested the correlations of the expression levels of EGFR and CDKN2A. As expected, compared with GBM patients without EGFR amplification, EGFR was highly expressed in GBM patients with EGFR amplification (Figure 3A). On the contrary, CKDN2A was highly expressed in GBM patients without EGFR amplification (Figure 3A). Also, with the deletion of CDKN2A in GBM patients, CDKN2A was downregulated, while EGFR was overexpressed (Figure 3B).

Next, all the genes regulated by EGFR amplification or CDKN2A deletion were identified. We found that 3,745 genes were differentially expressed in GBM patients with or without EGFR amplification, and 2,417 genes were differentially expressed in GBM patients with or without CDKN2A deletion. Moreover, 864 genes were commonly regulated by EGFR amplification and CDKN2A deletion (Figure 3C). Those genes classified GBM patients into two distinct subgroups, and each subgroup demonstrated different expression profiling and different genomic alterations (Figure 3D).

### EGFR Amplification and CDKN2A Deletion on Commonly Regulated Genes Are Highly Expressed in Brain Tissue and Associated With Cell Cycle, EBRR2, and MAPK Signaling Pathways

**Figure 4:**
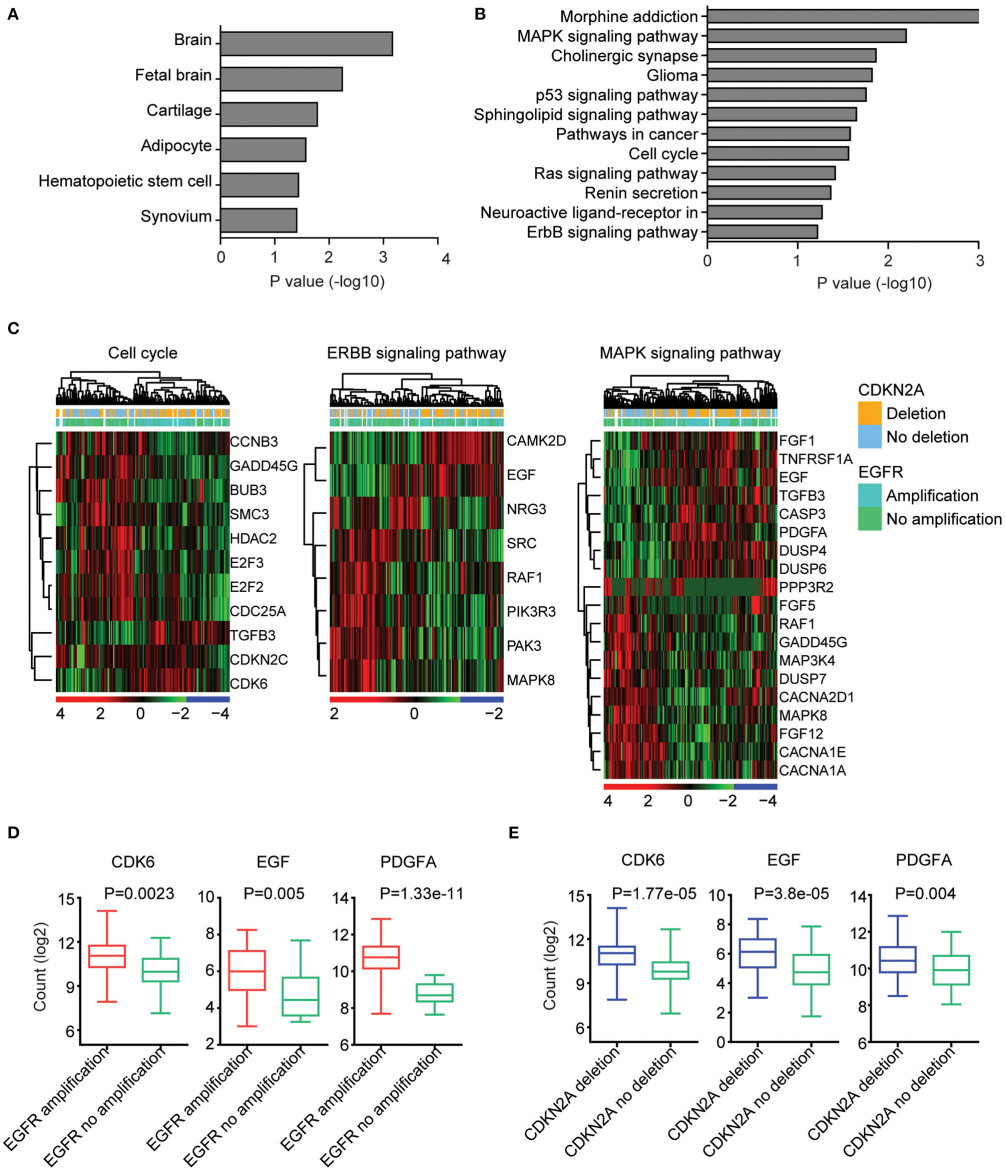
EGFR amplification and CDKN2A deletion on commonly regulated genes are highly expressed in brain tissue and associated with cell cycle, EBRR2, and MAPK signaling pathways. **(A)** Tissue enrichment analysis of the EGFR amplification and CDKN2A deletion on the commonly regulated genes using DAVID. The significantly enriched tissues were shown. **(B)** Functional pathway enrichment analysis of the EGFR amplification and CDKN2A deletion on the commonly regulated genes. The significantly enriched pathways were shown. **(C)** Unsupervised clustering heatmaps demonstrated the expression levels of EGFR amplification and CDKN2A deletion on the commonly regulated genes enriched in cell cycle, EBRR2, and MAPK signaling pathways. **(D)** Box plots showed the CDK6, EGF, and PDGFA expression levels (log_2_ normalization count) in TCGA GBM patients with (red) or without (green) EGFR amplification. *P*-values were performed using two-tailed paired Student's *t* test. **(E)** Box plots showed the CDK6, EGF, and PDGFA expression levels in TCGA GBM patients with (blue) or without (green) CDKN2A deletion.

We further determined the transcriptional relevance of EGFR amplification and CDKN2A deletion commonly regulated genes. We found that those genes were enriched in brain tissues (Figure 4A). Moreover, cell cycle, ERBB signaling pathway, and MAPK signaling pathway were associated with EGFR amplification and CDKN2A deletion (Figure 4B). The enriched EGFR amplification and CDKN2A deletion regulated genes in the cell cycle, ERBB, and MAPK signaling pathways were illustrated in the heatmaps (Figure 4C).

We further illustrated that CDK6 from the cell cycle, EGF from the ERBB signaling pathway, and PDGFA from the MAPK signaling pathway were all upregulated in GBM patients with EGFR amplification (Figure 4D). Also, CDK6, EGF, and PDGFA were all overexpressed in GBM patients with CDKN2A deletion (Figure 4E).

### *SPATS2L* Is Regulated by EGFR Amplification and CDKN2A Deletion and Associated With the Prognosis of GBM

**Figure 5:**
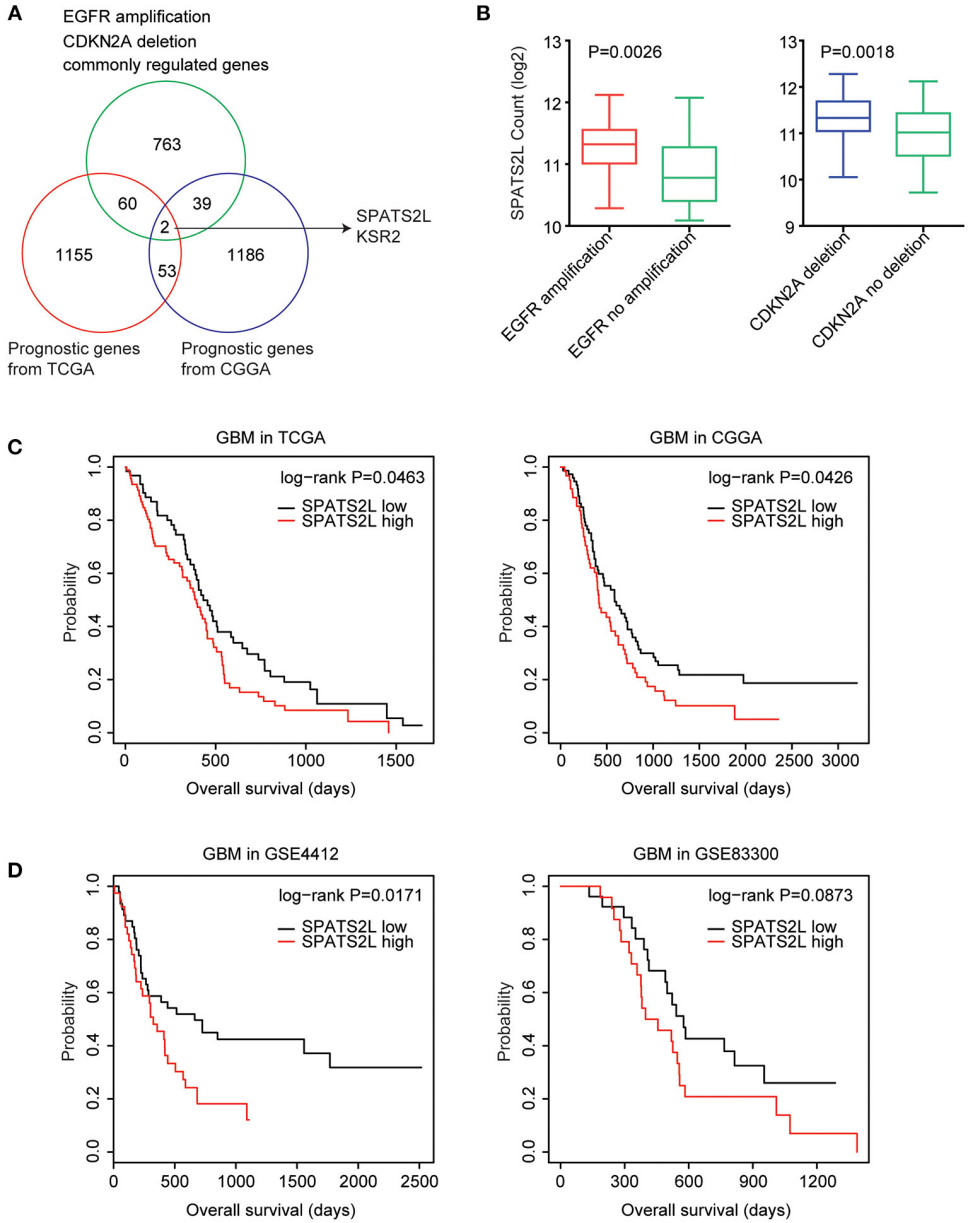
Spermatogenesis-associated serine-rich 2-like gene (*SPATS2L*) is regulated by EGFR amplification and CDKN2A deletion and associated with the prognosis of GBM. **(A)** Venn diagram depicted the prognostic effects on the commonly regulated genes by EGFR amplification and CDKN2A deletion. The two genes *SPATS2L* and *KSR2* were associated with the prognosis of GBM in both TCGA and CGGA datasets. **(B)** Box plots showed the *SPATS2L* expression levels (log_2_ normalization count) in TCGA GBM patients with (red) or without (green) EGFR amplification or GBM patients with (blue) or without (green) CDKN2A deletion. *P*-values were performed using two-tailed paired Student's *t*-test. **(C)** Relationships between *SPATS2L* expression levels and overall survival were analyzed in the TCGA and CGGA GBM datasets. Kaplan–Meier survival analysis was used to compare the overall survival of GBM patients with a high expression of *SPATS2L* (red) vs. GBM patients with a low expression of *SPATS2L* (black). *P* values were generated from the log-rank test. **(D)** The Kaplan–Meier plots showed the clinical outcomes of GBM patients with high *SPATS2L* expression (red) or low *SPATS2L* expression (black) in the GSE4412 and GSE83300 datasets. *P*-values were generated from the log-rank test.

Next, we determined the prognostic relevance of EGFR amplification and CDKN2A deletion on commonly regulated genes. Among the 864 genes commonly regulated by EGFR amplification and CDKN2A deletion, 62 genes were significantly associated with the clinical outcomes of GBM derived from the TCGA GBM datasets (Figure 5A). Also, using the CGGA datasets, we found that 41 genes out of the 864 genes demonstrated prognostic effects in GBM (Figure 5A). Interestingly, the two genes *SPATS2L* and *Kinase suppressor of ras 2* (*KSR2*) were both significantly correlated with the prognosis of GBM in the TCGA and CGGA datasets (Figure 5A).

*SPATS2L* was upregulated in GBM patients with EGFR amplification, compared with GBM patients without EGFR amplification (Figure 5B). Also, compared with GBM patients without CDKN2A deletion, the expression levels of *SPATS2L* were higher in patients with CDNK2A deletion (Figure 5B). Correspondingly, the higher expression levels of *SPATS2L* were associated with worse prognosis in patients with GBM in both TCGA and CGGA datasets (Figure 5C).

The prognostic effects of *SPATS2L* were further validated using the GEO datasets. In the GSE4412 dataset, we found that the higher expression levels of *SPATS2L* were associated with worse prognosis in patients with GBM (*P* = 0.01) (Figure 5D). However, GBM patients with higher expression levels of *SPATS2L* had no significant worse clinical outcomes in the GSE83300 dataset (*P* = 0.087) (Figure 5D). Those results highlighted that *SPATS2L* was an important prognostic marker in patients with GBM.

*KSR2* was specifically expressed in brain tissues (33). We found that *KSR2* was downregulated in GBM patients with EGFR amplification or GBM patients with CDNK2A deletion (Supplementary Figure 3A). However, we found the contradictive prognostic effects of *KSR2* in the TCGA and CGGA datasets. The higher expression levels of *KSR2* were associated with worse prognosis in patients with GBM in the TCGA datasets, while *KSR2* was a good prognostic marker in the CGGA datasets (Supplementary Figure 3B). So, *KSR2* was not further studied.

### Expression Levels of *SPATS2L* in Different Subtypes of Patients With GBM

**Figure 6:**
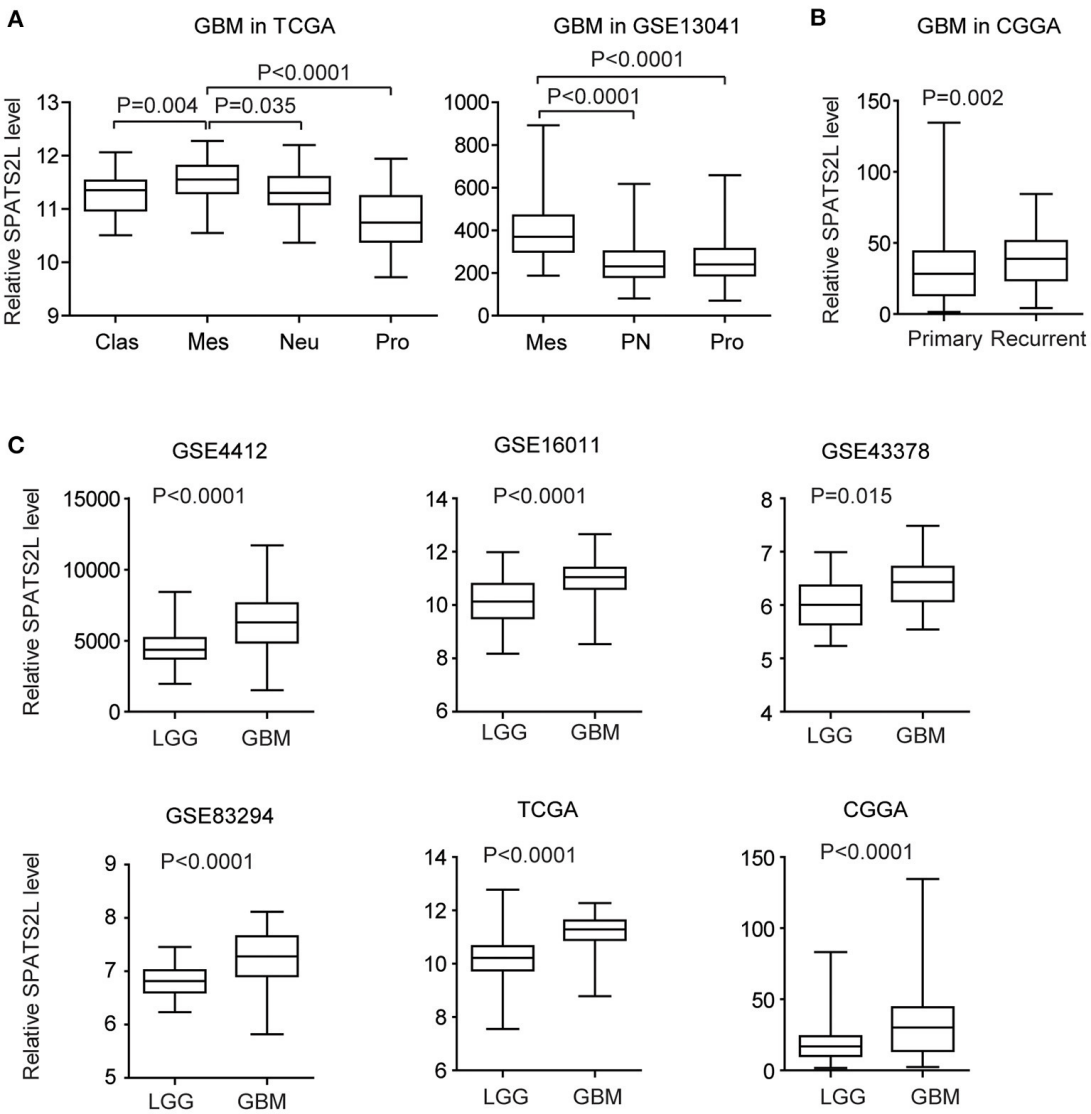
Expression levels of *SPATS2L* in different subtypes of patients with GBM. **(A)** Box plots demonstrated the expression levels of *SPATS2L* in different GBM subtypes in the TCGA and GSE13041 datasets. *P*-values were performed using two-tailed paired Student's *t*-test. Clas, classical; Neu, neural; Pro, proneural; Mes, mesenchymal; PN, proneural; Pro, proliferative. **(B)** Box plots demonstrated the expression levels of *SPATS2L* in GBM primary tissues and recurrent tissues in the CGGA datasets. **(C)** Box plots demonstrated the expression levels of *SPATS2L* in GBM and lower grade glioma (LGG) patients in the GSE4412, GSE16011, GSE43378, GSE83294, TCGA, and CGGA datasets.

GBM is divided into four subtypes by the TCGA GBM research group: classical (Clas), neural (Neu), proneural (Pro), and mesenchymal (Mes) (37). The present study then assessed the expression levels of the *SPATS2L* in patients with different subtypes of GBM. Compared with the Clas, Neu, and Pro subtypes, the expression levels of *SPATS2L* were higher in patients with the Mes subtype of GBM in the TCGA datasets (Figure 6A). GBM was also classified into three subtypes—proneural (PN), mesenchymal (Mes), and proliferative (Pro)—based on their molecular signatures (38). This higher expression of the *SPATS2L* in the Mes subtype of GBM patients was also observed in the GSE13041 dataset, compared with the PN and Pro subtypes (Figure 6A). Also, compared with primary GBM tissues, *SPATS2L* was highly expressed in recurrent GBM tissues in the CGGA datasets (Figure 6B).

GBM is grade IV glioma. We found that, compared with grade II–III glioma (LGG), the expression levels of *SPATS2L* were higher in GBM patients in the GSE4412, GSE16011, GSE43378, GSE83294, TCGA, and CGGA datasets (Figure 6C). Those results suggested the potential prognostic significance of *SPATS2L* in patients with LGG.

### Coordinated Prognostic Relevance and Co-occurrence of EGFR Amplification and CDKN2A Deletion in Patients With LGG

**Figure 7:**
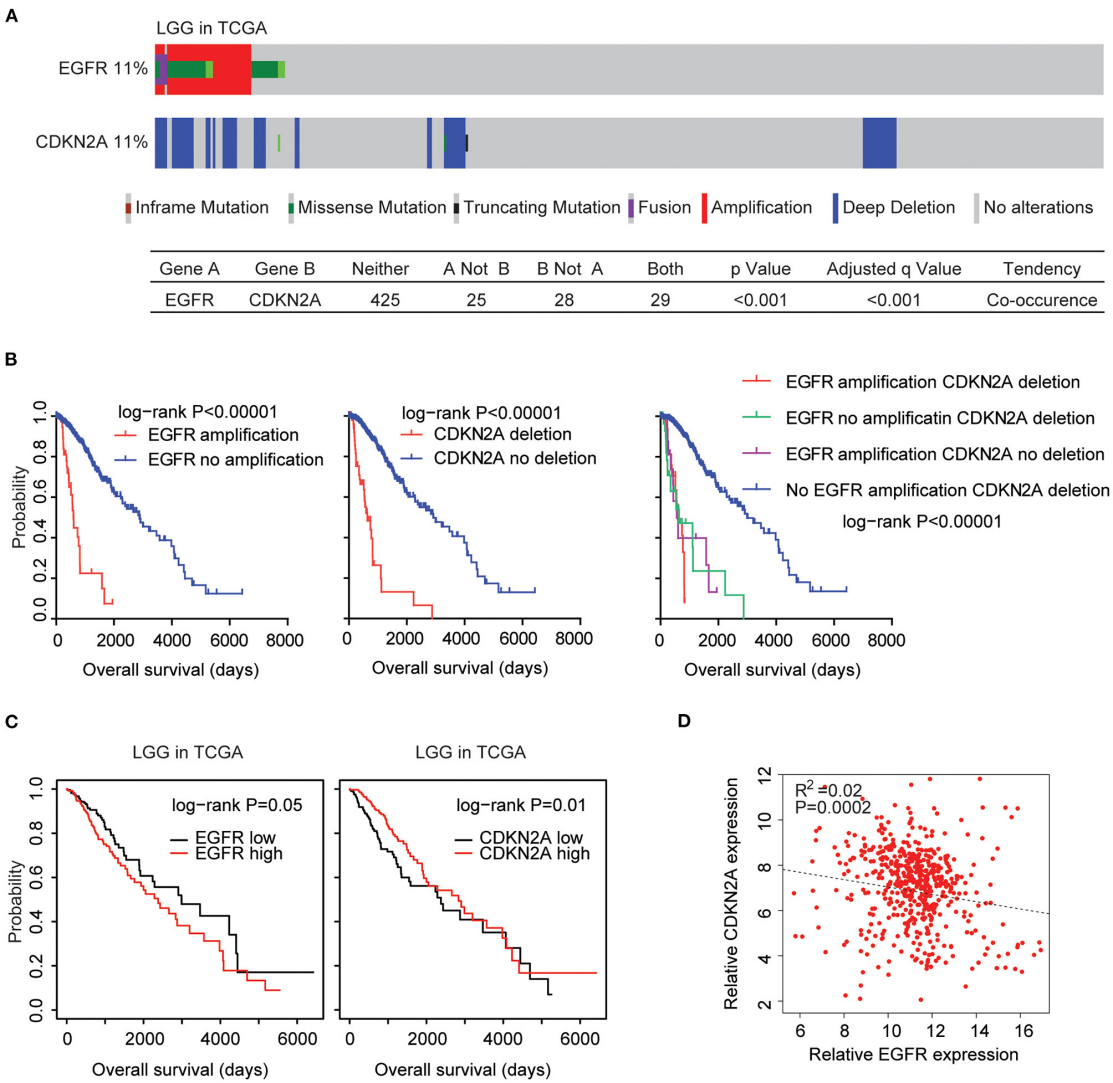
Alteration coexistence and prognostic relevance of EGFR amplification and CDKN2A deletion in patients with LGG. **(A)** OncoPrint demonstrated the co-occurrence of EGFR alteration and CDKN2A deletion in patients with LGG derived from the TCGA datasets. Red indicated gene amplification, blue indicated gene deletion, and green represented gene mutation. Each line represented one patient. **(B)** The Kaplan–Meier plot demonstrated the prognostic effects of EGFR amplification and CDKN2A deletion in patients with LGG in the TCGA datasets. The log-rank test was used to determine the different overall survival of patients with (red) or without (blue) EGFR amplification or patients with (red) or without (blue) CKDN2A deletion or patients with different alterations of EGFR and CDKN2A. *P*-values were generated from the log-rank test. **(C)** Kaplan–Meier survival analysis was used to compare the overall survival of LGG patients with a high expression of EGFR or CDKN2A (red) vs. LGG patients with a low expression of EGFR or CDKN2A (black). *P*-values were generated from the log-rank test. **(D)** Spearman correlations between EGFR and CDKN2A expression in LGG patients were derived from the TCGA datasets.

The EGFR and CDKN2A alteration frequency was relatively lower in patients with LGG (Figure 1A). Among the 507 LGG patients found in the TCGA datasets, 11% patients had EGFR alteration and 11% patients had CDKN2A deletion. Interestingly, 29 LGG patients had both EGFR and CDKN2A alterations. Statistical analysis showed that the coexistence of EGFR alteration and CDKN2A deletion was significant in patients with LGG (*P* < 0.0001) (Figure 7A).

Next, we tested the prognostic effects of EGFR amplification and CDKN2A deletion in LGG patients. We found that LGG patients with EGFR amplification demonstrated worse prognosis compared with patients without EGFR amplification (*P* < 0.0001) (Figure 7B). Similarly, LGG patients with CDKN2A deletion also had worse prognosis compared with patients without CDKN2A deletion (*P* < 0.0001) (Figure 7B). Moreover, LGG patients without EGFR amplification and CDKN2A deletion had the best prognosis than LGG patients with EGFR amplification or CDKN2A deletion or with both alterations (Figure 7B). All those results emphasized the associations of EGFR amplification and CDKN2A deletion in determining the clinical outcomes of LGG patients.

However, unlike GBM patients, the higher expression levels of EGFR were associated with worse prognosis in patients with LGG in the TCGA LGG datasets (Figure 7C). On the contrary, the lower expression levels of CDKN2A were associated with worse prognosis in patients with LGG (Figure 7C). Spearman correlation demonstrated high negative correlations between EGFR and CDKN2A expression in the TCGA LGG datasets (Figure 7D).

**Figure 8:**
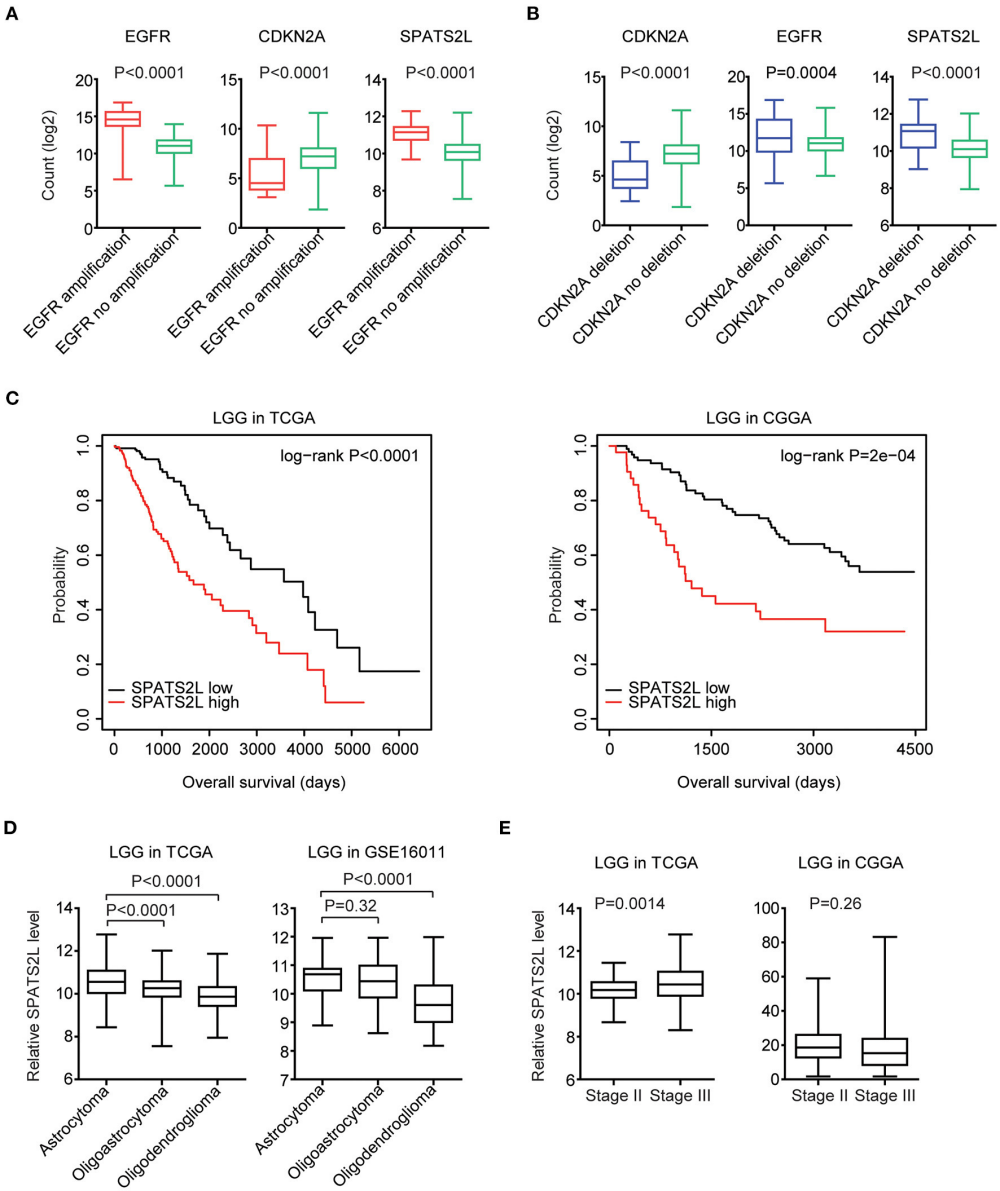
Prognostic significance of *SPATS2L* in patients with LGG. **(A)** Box plots showed the EGFR, CDKN2A, and *SPATS2L* expression levels (log_2_ normalization count) in TCGA LGG patients with (red) or without (green) EGFR amplification. *P*-values were performed using Student's *t*-test. **(B)** Box plots showed the EGFR, CDKN2A, and *SPATS2L* expression levels in TCGA LGG patients with (blue) or without (green) CDKN2A deletion. **(C)** Kaplan–Meier survival analysis was used to compare the overall survival of LGG patients with a high expression of *SPATS2L* (red) vs. LGG patients with a low expression of *SPATS2L* (black) in the TCGA and CGGA datasets. *P*-values were generated from the log-rank test. **(D)** Box plots demonstrated the expression levels of *SPATS2L* in different LGG subtypes. *P*-values were performed using Student's *t*-test. **(E)** Box plots demonstrated the expression levels of *SPATS2L* in LGG primary tissues and recurrent tissues.

Moreover, in LGG patients with EGFR amplification, EGFR was highly expressed. However, CKDN2A was downregulated in LGG patients with EGFR amplification (Figure 8A). Also, compared with LGG patients without CDKN2A deletion, CDKN2A was downregulated, and EGFR was overexpressed in LGG patients with CDKN2A deletion (Figure 8B).

### Prognostic Significance of *SPATS2L* in Patients With LGG

Since EGFR and CDKN2A alterations and expression levels were all associated with the clinical outcomes of patients with LGG, we then tested the prognostic effects of EGFR and CDKN2A alterations on the regulated gene *SPATS2L*. *SPATS2L* was upregulated in LGG patients with EGFR amplification, compared with LGG patients without EGFR amplification (Figure 8A). Also, compared with LGG patients without CDKN2A deletion, the expression levels of *SPATS2L* were relatively higher in patients with CDKN2A deletion (Figure 8B). Moreover, the higher expression levels of *SPATS2L* were associated with worse prognosis in patients with LGG in both TCGA and CGGA datasets (Figure 8C).

We also assessed the expression levels of *SPATS2L* in patients with different subtypes of LGG. LGG was divided into astrocytoma, oligoastrocytoma, and oligodendroglioma subtypes (3). Compared with the oligoastrocytoma and oligodendroglioma subtypes, the expression levels of *SPATS2L* were higher in patients with the astrocytoma subtype of LGG in the TCGA datasets (Figure 8D). The high expression levels of *SPATS2L* in the astrocytoma subtype of LGG patients were also observed in the GSE16011 dataset, compared with the oligodendroglioma subtype (Figure 8D). The present study also assessed the expression levels of *SPATS2L* in different stages of patients with LGG. Compared with stage II LGG, *SPATS2L* was highly expressed in patients with stage III LGG in the TCGA datasets (Figure 8E). However, there were no significantly different expression levels of *SPATS2L* in different stages of LGG patients in the CGGA datasets (Figure 8E).

### Validation of the Genetic Connections of EGFR and CDKN2A Alteration Using the MSKCC Datasets

**Figure 9:**
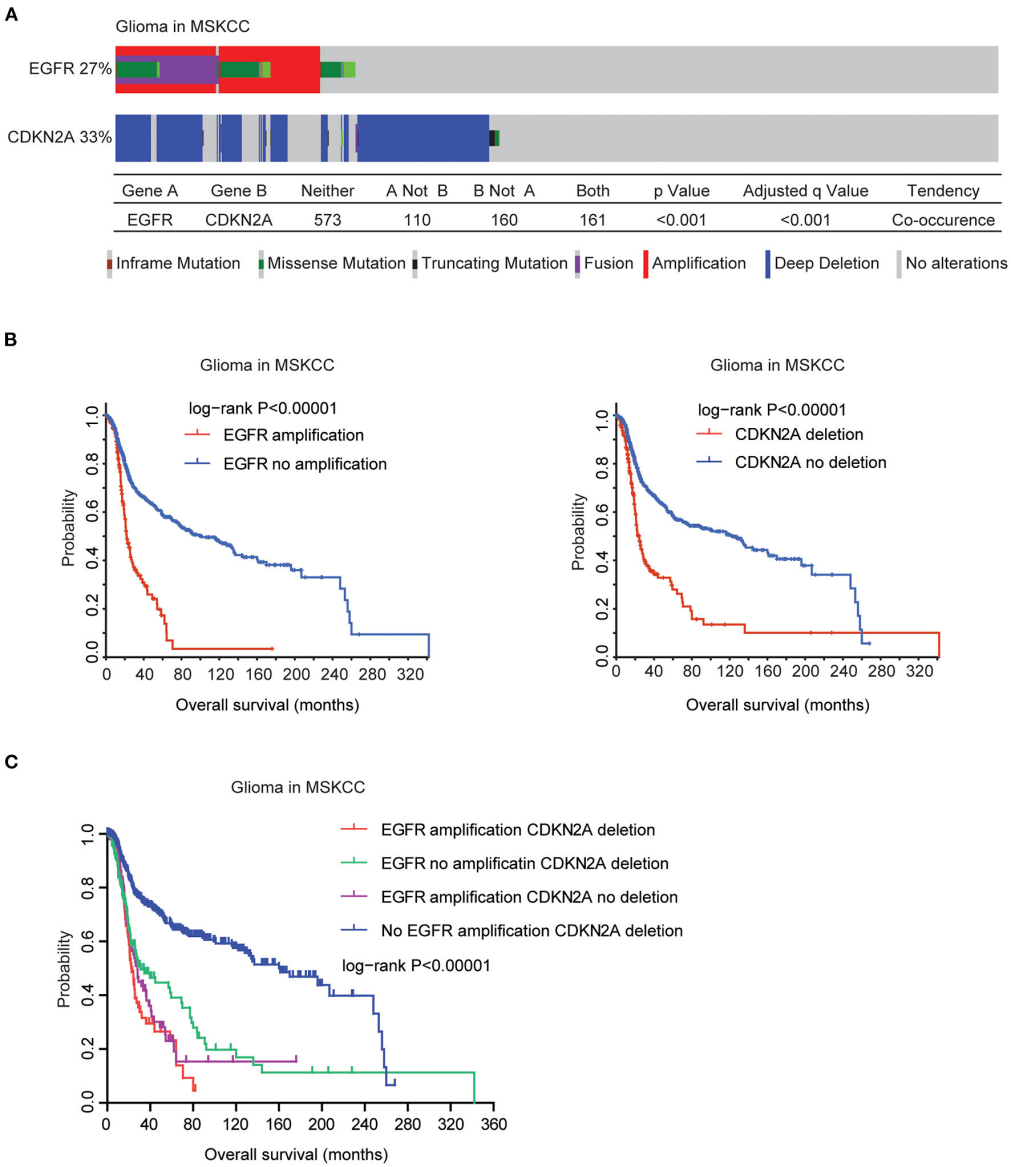
Validation of the genetic connections of EGFR and CDKN2A alterations using the MSKCC datasets. **(A)** OncoPrint demonstrated the co-occurrence of EGFR alteration and CDKN2A deletion in glioma patients derived from the MSKCC datasets. (**B**) The Kaplan–Meier plot demonstrated the prognostic effects of EGFR amplification and CDKN2A deletion in glioma patients from the MSKCC datasets. **(C)** The Kaplan–Meier plot demonstrated the different overall survival of glioma patients with different alterations of EGFR and CDKN2A. *P*-values were generated from the log-rank test.

Finally, we validated the genetic connections and the prognostic significance of EGFR and CDKN2A alterations using the MSKCC glioma datasets (39). Similarly, in the MSKCC datasets, EGFR alteration was coexistent with CDKN2A deletion. Among the 1,004 glioma patients in the MSKCC datasets, 27% patients had EGFR alteration and 33% patients had CDKN2A deletion. One hundred and sixty-one glioma patients had both EGFR and CDKN2A alterations (Figure 9A).

Moreover, in the MSKCC datasets, EGFR amplification was also associated with bad prognosis of glioma. Glioma patients without EGFR amplification had longer overall survival (Figure 9B). Similarly, glioma patients without CDKN2A deletion also had better prognosis, compared with glioma patients with CDKN2A deletion (Figure 9B). Furthermore, glioma patients without EGFR amplification and CDKN2A deletion had the best prognosis than glioma patients with EGFR amplification or CDKN2A deletion or with both alterations in the MSKCC datasets (Figure 9C). Those results were consistent with previous results derived from the TCGA datasets.

## Discussion

Cancer development requires multiple somatic alterations which contribute to selective advantages in the malignant transformation of cancer cells. Understanding the interactions of those genetic alterations is critical to cancer treatment. For example, MYC is mutually exclusive with PIK3CA, PTEN, APC, or BRAF alterations (40), suggesting that MYC is a distinct driver gene. EGFR mutation and CDKN2A deletion co-occurred in a subgroup of lung cancer and were associated with the clinical outcome of EGFR inhibitor treatment (31). Here, we revealed the co-occurrence of EGFR amplification and CDKN2A deletion in patients with glioma, suggesting that the poor drug response of EGFR inhibitors in glioma may be associated with CDKN2A deletion.

EGFR amplification and CDKN2A deletion were also associated with the clinical overall survival of GBM. Moreover, EGFR amplification and CDKN2A deletion on the commonly regulated gene *SPATS2L* were also associated with the prognosis and sub-classification of patients with GBM. The prognostic effects of *SPATS2L* were never reported. Those new discoveries highlighted the significance of the TCGA, CGGA, and MSKCC datasets which could be used to identify new factors to predict the clinical outcomes of patients with GBM.

LGG and GBM are different grades of glioma with different clinical outcomes and molecular profiling (4, 5). Compared with GBM, EGFR amplification and CDKN2A deletion happened less frequently in LGG patients. However, like GBM, EGFR amplification and CDKN2A deletion co-occurred in LGG patients and in the commonly regulated *SPATS2L* expression. Moreover, *SPATS2L* was associated with the prognosis and sub-classification of patients with LGG. Those results emphasized some common molecular profiling of GBM and LGG and suggested that some biomarkers could be used in both GBM and LGG patients.

Overall, our results provide further understanding of how EGFR amplification and CDKN2A deletion influence the clinical overall survival of GBM and LGG patients. Although further clinical validations are needed, our analysis suggests that EGFR, CDKN2A status, and the expression levels of *SPATS2L* could be used as biomarkers to predict the overall survival of GBM and LGG patients.

## Data Availability Statement

Publicly available datasets were used in this study. The TCGA gene expressions along with the clinical datasets were downloaded from the TCGA hub (https://tcga.xenahubs.net). The CGGA datasets were available at http://www.cgga.org.cn/index.jsp website. The gene expression series matrix of glioma tissues was downloaded from the GEO website (www.ncbi.nlm.nih.gov/geo), including GSE4412, GSE83300, GSE16011, GSE43378, GSE83294, and GSE16011 datasets.

## Author Contributions

HW designed the study and wrote the manuscript. HW, XW, and LX performed the data analysis. HC and JZ designed the study and supervised the work. All authors contributed to the article and approved the submitted version.

## Conflict of Interest

The authors declare that the research was conducted in the absence of any commercial or financial relationships that could be construed as a potential conflict of interest.

## Supplementary Material

Supplementary Figure 1 Alteration frequencies and prognostic relevance of EGFR mutations across 32 tumor types. **(A)** Percentage of cancer patients with EGFR mutations across 32 tumor types. Each line represented one type of tumor. **(B)** Kaplan-Meier plot demonstrated the prognostic effects of EGFR mutations across 32 tumor types of patients in TCGA datasets. The log-rank test was used to determine the different overall survival of tumor patients with (red) or without (blue) EGFR mutations. **(C)** Kaplan-Meier plot demonstrated the prognostic effects of EGFR mutations in patients with GBM in TCGA datasets.

Supplementary Figure 2 Prognostic relevance of EGFR and CDKN2A in patients with GBM. Kaplan-Meier survival analysis was used to reveal the prognostic relevance of EGFR and CDKN2A expression levels in TCGA datasets. *P*-values were generated from Log-rank test.

Supplementary Figure 3 Expression and prognostic relevance of KSR2 in patients with GBM. **(A)** Box plots showed the KSR2 expression levels (log_2_ normalization count) in TCGA LGG patients with or without EGFR or CDKN2A alterations. *P*-values were performed using Student's *t*-test. **(B)** Kaplan-Meier survival analysis was used to compare the overall survival of KSR2 highly expressed GBM patients (red) with KSR2 lowly expressed GBM patients (black) in TCGA and CGGA datasets. *P*-values were generated from Log-rank test.
