# Supplementary figures and images for: Analysis of the EGFR Amplification and CDKN2A Deletion Regulated Transcriptomic Signatures Reveals the Prognostic Significance of SPATS2L in Patients With Glioma
Source: Front Oncol. 2021 Apr 20;11:551160. doi: 10.3389/fonc.2021.551160 (PMC8093400; doi:10.3389/fonc.2021.551160)

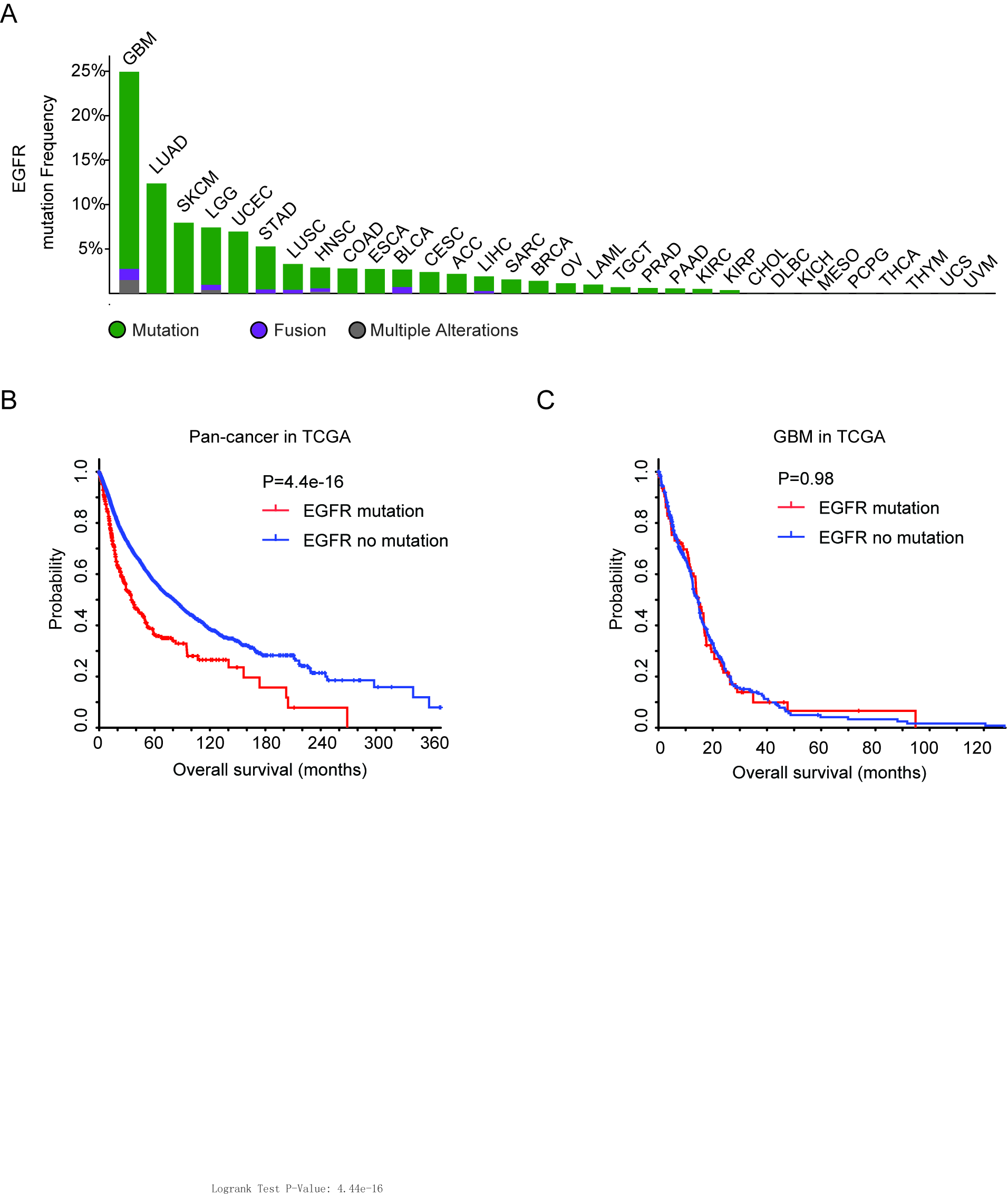

Supplement: Supplementary Figure 1 — Alteration frequencies and prognostic relevance of EGFR mutations across 32 tumor types. (A) Percentage of cancer patients with EGFR mutations across 32 tumor types. Each line represented one type of tumor. (B) Kaplan-Meier plot demonstrated the prognostic effects of EGFR mutations across 32 tumor types of patients in TCGA datasets. The log-rank test was used to determine the different overall survival of tumor patients with (red) or without (blue) EGFR mutations. (C) Kaplan-Meier plot demonstrated the prognostic effects of EGFR mutations in patients with GBM in TCGA datasets. [file Image_1.TIF]

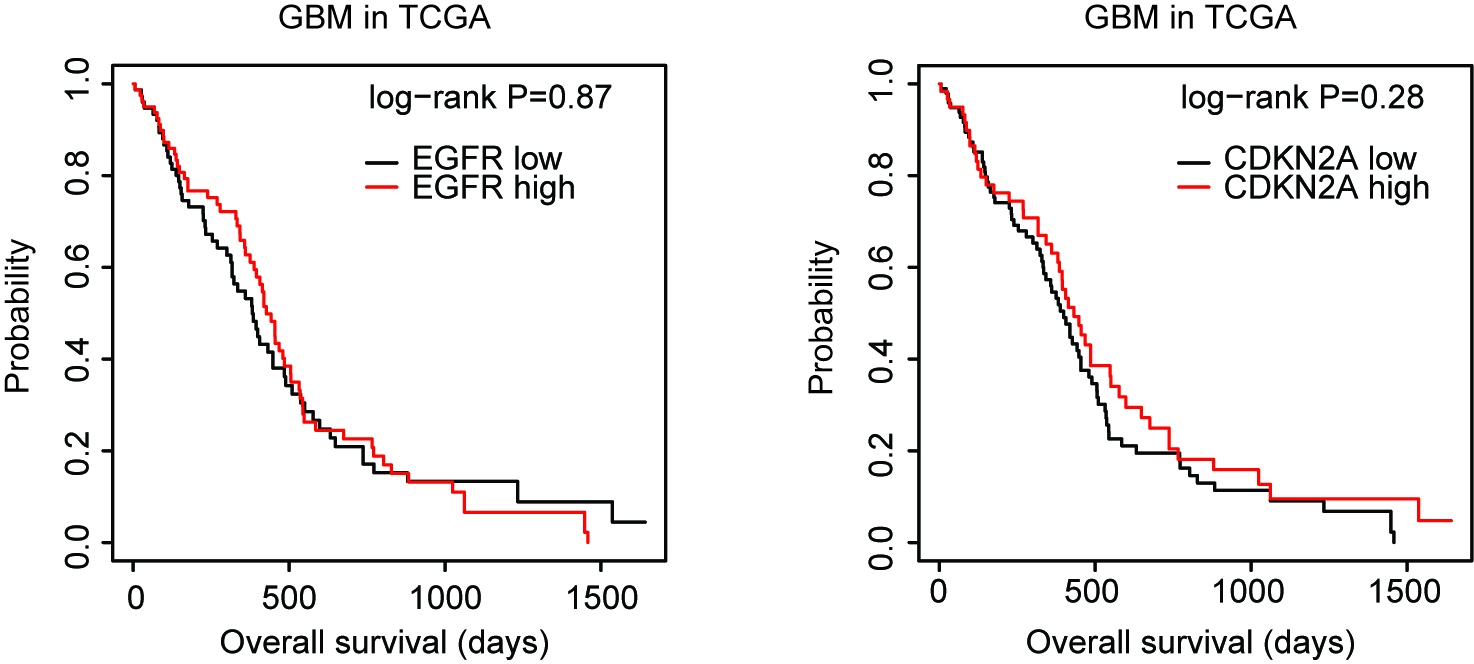

Supplement: Supplementary Figure 2 — Prognostic relevance of EGFR and CDKN2A in patients with GBM. Kaplan-Meier survival analysis was used to reveal the prognostic relevance of EGFR and CDKN2A expression levels in TCGA datasets. P-values were generated from Log-rank test. [file Image_2.TIF]

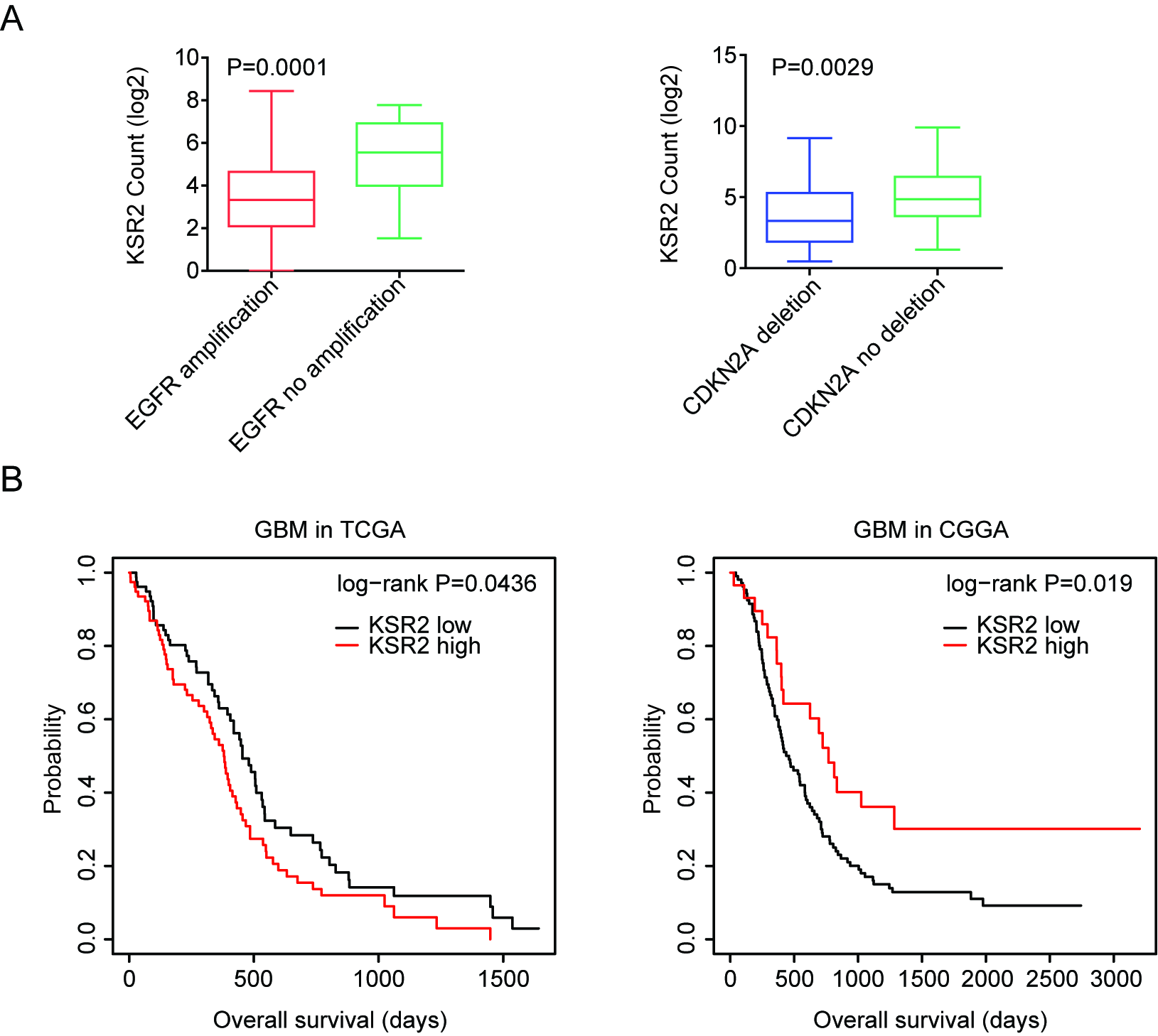

Supplement: Supplementary Figure 3 — Expression and prognostic relevance of KSR2 in patients with GBM. (A) Box plots showed the KSR2 expression levels (log2 normalization count) in TCGA LGG patients with or without EGFR or CDKN2A alterations. P-values were performed using Student's t-test. (B) Kaplan-Meier survival analysis was used to compare the overall survival of KSR2 highly expressed GBM patients (red) with KSR2 lowly expressed GBM patients (black) in TCGA and CGGA datasets. P-values were generated from Log-rank test. [file Image_3.TIF]
